# Acute Synovitis—Incision into the Joint

**Published:** 1871-10

**Authors:** 


Acute Synovitis—Incision into the Joint.—Mr. J. R. Jessop, F. R. C. S., in a lecture published by the *British Medical Journal*, states that he lately successfully followed Prof. Lister's plan, and incised into the knee joint of a patient aged twenty years, who suffered from acute synovitis, after the ordinary treatment adopted in such cases had been tried, *i. e.*, rest, leeches, ice, evaporating lotions, salines, etc., etc. Mr. Jessop made an opening into the joint, and in the axis of the thigh, commencing one inch above the patella, the opening was an inch long, but had to be enlarged to one and a half inches to allow flakes of lymph to pass through, which were suspended in from eight to ten ounces of clear fluid. From the time the incision was made the excruciating pain ceased, the fever disappeared, the swelling never returned, and the patient was sent from the Leeds Infirmary to a convalescent hospital with a movable painless joint within a month from the time of the operation.— *The Doctor*.

